# Aspects and Implementation of Pharmaceutical Quality by Design from Conceptual Frameworks to Industrial Applications

**DOI:** 10.3390/pharmaceutics17050623

**Published:** 2025-05-08

**Authors:** Shiwei Yang, Xingming Hu, Jinmiao Zhu, Bin Zheng, Wenjie Bi, Xiaohong Wang, Jialing Wu, Zimeng Mi, Yifei Wu

**Affiliations:** 1 Department of Chemistry and Chemical Engineering, Hefei Normal University, Hefei 230061, China; 2 State Key Laboratory of Drug Research, Shanghai Institute of Materia Medica, Chinese Academy of Sciences, Shanghai 201203, China; 3 Anhui Provincial Engineering Laboratory for the Development and Utilization of Medicinal and Edible Natural Resources, Hefei Normal University, Hefei 230061, China

**Keywords:** quality by design, design of experiments, drug development

## Abstract

**Background/Objectives:** Quality by Design (QbD) has revolutionized pharmaceutical development by transitioning from reactive quality testing to proactive, science-driven methodologies. Rooted in ICH Q8–Q11 guidelines, QbD emphasizes defining Critical Quality Attributes (CQAs), establishing design spaces, and integrating risk management to enhance product robustness and regulatory flexibility. This review critically examines QbD’s theoretical frameworks, implementation workflows, and industrial applications, aiming to bridge academic research and commercial practices while addressing emerging challenges in biologics, advanced therapies, and personalized medicine. **Methods:** The review synthesizes regulatory guidelines, case studies, and multidisciplinary tools, including Design of Experiments (DoE), Failure Mode Effects Analysis (FMEA), Process Analytical Technology (PAT), and multivariate modeling. It evaluates QbD workflows—from Quality Target Product Profile (QTPP) definition to control strategies—and explores advanced technologies like AI-driven predictive modeling, digital twins, and continuous manufacturing. **Results:** QbD implementation reduces batch failures by 40%, optimizes dissolution profiles, and enhances process robustness through real-time monitoring (PAT) and adaptive control. However, technical barriers, such as nonlinear parameter interactions in complex systems, and regulatory disparities between agencies hinder broader adoption. **Conclusions:** QbD significantly advances pharmaceutical quality and efficiency, yet requires harmonized regulatory standards, lifecycle validation protocols, and cultural shifts toward interdisciplinary collaboration. Emerging trends, including AI-integrated design space exploration and 3D-printed personalized medicines, promise to address scalability and patient-centric needs. By fostering innovation and compliance, QbD remains pivotal in achieving sustainable, patient-focused drug development.

## 1. Introduction

### 1.1. Historical Context of Pharmaceutical Quality Control

Traditional pharmaceutical quality control (QC) historically relied on end-product testing and empirical “trial-and-error” development approaches, which introduced significant limitations. Endpoint testing, such as high-performance liquid chromatography (HPLC) or dissolution testing, focused solely on verifying compliance with predefined specifications for final products, offering no proactive insights into process variability or root cause analysis of defects [1]. This reactive model often led to batch failures, recalls, and regulatory non-compliance due to insufficient understanding of critical quality attributes (CQAs) and process parameters (CPPs). CQAs define critical quality targets (e.g., dissolution rate), while CPPs are process parameters (e.g., compression force) requiring precise control to ensure product consistency and compliance with predefined specifications. CQA example: dissolution rate of a tablet (e.g., “≤80% active pharmaceutical ingredient released within 30 min in pH 6.8 medium”)—a direct measure of bioavailability and therapeutic efficacy, validated against pharmacopeial standards (e.g., USP/Ph. Eur.). CPP example: compression force during tablet manufacturing (e.g., “controlled within 10–15 kN”)—directly impacts tablet hardness, porosity, and dissolution performance, with deviations leading to friability failures or altered release profiles. Additionally, empirical development methods lacked systematic scientific rationale, resulting in rigid processes resistant to scaling or optimization. For instance, formulation or manufacturing changes necessitated costly revalidation, hindering innovation and efficiency [2]. Such approaches inadequately addressed the complexity of modern biopharmaceuticals and failed to align with evolving regulatory expectations for risk-based quality assurance.

The International Council for Harmonisation (ICH) Q8–Q11 guidelines marked a paradigm shift toward Quality by Design (QbD), emphasizing proactive quality management through science- and risk-based methodologies. ICH Q8 (Pharmaceutical Development) introduced the concept of design space, enabling flexible manufacturing within predefined multivariate parameter ranges, while Q9 (Quality Risk Management) formalized risk assessment tools to prioritize CQAs and CPPs [3]. Q10 (Pharmaceutical Quality System) and Q11 (Development and Manufacture of Drug Substances) further integrated continuous improvement and control strategies, replacing static specifications with dynamic, lifecycle-oriented approaches [4]. Regulatory agencies, including the FDA and EMA, championed QbD through initiatives like Process Analytical Technology (PAT), incentivizing real-time monitoring and data-driven decision-making [5]. This evolution was driven by the need to reduce post-market failures, enhance manufacturing agility, and align global standards. By embedding quality into product design rather than relying on retrospective testing, QbD has redefined pharmaceutical development, fostering innovation while ensuring patient safety [6].

### 1.2. Definition and Core Principles of QbD

QbD is formally defined by the ICH Q8(R2) guideline as “a systematic approach to development that begins with predefined objectives and emphasizes product and process understanding and process control, based on sound science and quality risk management” [7]. This definition underscores QbD as a proactive framework aimed at embedding quality into pharmaceutical products through deliberate design, rather than relying solely on end-product testing. Central to QbD is the establishment of a design space, a multidimensional region of input variables (e.g., material attributes, process parameters) proven to ensure product quality, as defined by critical quality attributes (CQAs) [7,8]. The design space is derived from experimental data and mechanistic models, enabling flexible manufacturing within regulatory-approved boundaries [9]. By prioritizing science- and risk-based methodologies, QbD integrates tools like risk assessment (e.g., Failure Mode Effects Analysis, FMEA) and Design of Experiments (DoE) to identify and mitigate variability sources early in development [10].

The core principles of QbD extend to control strategies and continuous improvement. A control strategy, as per ICH Q10, encompasses planned controls (e.g., in-process monitoring, procedural controls) to ensure consistent product quality within the design space [11]. These controls are dynamically adjusted using real-time data from advanced process analytical technologies (PAT), aligning with the principle of continuous improvement [12]. For instance, multivariate data analysis and machine learning enable ongoing process optimization, reducing deviations and enhancing robustness [13]. Regulatory agencies, including the FDA, endorse QbD for its ability to harmonize innovation with quality assurance, fostering a lifecycle approach to pharmaceutical manufacturing [14]. Collectively, QbD transforms quality from a static endpoint to an evolving target, grounded in scientific rigor and adaptive risk management.

### 1.3. Objectives of the Review

The primary objective of this review is to systematically dissect the theoretical framework and implementation pathways of QbD in pharmaceutical sciences. Rooted in the ICH guidelines Q8–Q12, QbD advocates a science-driven, risk-based methodology to ensure product quality through predefined CQAs, Critical Material Attributes (CMAs), and Critical Process Parameters (CPPs) [7,15]. Central to this framework is the establishment of a design space, a multidimensional combination of variables that ensures robust product performance, as defined by ICH Q8(R2) [10,16]. This review evaluates the integration of tools such as risk assessment matrices (e.g., Failure Mode and Effects Analysis, FMEA), DoE, and mechanistic modeling to bridge the gap between empirical development and predictive control references to Table 1 (detailed in Table 1). For instance, Nandi, U. et al. (2021) demonstrated how multivariate analysis optimizes QbD-driven processes by linking material variability to CQAs in solid dosage forms [17]. Additionally, the role of PAT, as endorsed by the U.S. FDA’s Process Validation Guidance (2011), is scrutinized for its capacity to enable real-time monitoring and adaptive control, thereby reducing batch failures [18].

A secondary objective is to critically address the practical challenges of QbD application across formulation development, process optimization, and lifecycle management. Despite its theoretical rigor, QbD implementation faces obstacles such as incomplete characterization of complex drug formulations (e.g., biologics, nanomedicines) and nonlinear interactions in multiphase systems references to Table 1 (as summarized in Table 1). For example, Szabó E. et al. (2019) highlighted limitations in predictive models for amorphous solid dispersions, where kinetic instability and phase separation undermine design space reliability [19]. In biopharmaceutical manufacturing, Finkler C. et al. (2020) identified scalability issues due to equipment heterogeneity and raw material drift, particularly in monoclonal antibody production [20]. Lifecycle management under QbD, as outlined in ICH Q12, demands continuous process verification and dynamic control strategies, yet organizational resistance to iterative regulatory submissions and data fragmentation in quality management systems (QMS) remain persistent barriers [21]. Emerging solutions, such as machine learning algorithms for sensitivity analysis (Jain, N. et al., 2024) and digital twin technologies for real-time simulation, are discussed as potential mitigants to these challenges [22].

### 1.4. QbD Core Concepts for Development and Product Design to Achieve Quality Frontiers by Methodologies and Tools Employed

Quality by Design (QbD), pioneered by thought leaders such as Joseph M. Juran and Genichi Taguchi, is rooted in the principle of proactively embedding quality into products during the design phase (“off-line quality”) rather than relying on post hoc corrections. This systematic framework asserts that quality is not an outcome of inspection but a consequence of deliberate planning, risk mitigation, and predictive modeling. Central to QbD is its holistic view of quality, which spans four interdependent dimensions: quality of design (specification alignment), quality of production (process consistency), quality of use (user-centric performance), and quality of post-sales relationships (sustained customer satisfaction). While QbD addresses all four components, its primary focus lies in the design phase, where conceptual development (e.g., stakeholder requirements, regulatory constraints) is rigorously integrated with product design (e.g., material selection, process parameter optimization) to achieve “quality by intention”.

Modern quality standards, such as ISO, reinforce this philosophy by mandating structured planning during early conceptualization, thereby institutionalizing QbD’s role in mitigating lifecycle risks. Furthermore, robust design—a cornerstone of QbD—relies on methodologies such as Design of Experiments (DoE) and Failure Mode and Effects Analysis (FMEA) to minimize variability and enhance product resilience under diverse operating conditions. By leveraging these tools, QbD ensures that design choices are data-driven, scalable, and aligned with predefined Critical Quality Attributes (CQAs), ultimately bridging the gap between theoretical innovation and market-ready excellence.

## 2. Key Elements of QbD Implementation

### 2.1. Target Product Quality Profile (TPQP)

The transition from QTPP (Quality Target Product Profile) to TPQP (Target Product Quality Profile) reflects an expanded scope emphasizing patient-centricity and lifecycle adaptability. TPQP integrates contemporary elements such as patient-reported outcomes (PROs), real-world evidence (RWE), and regulatory frameworks like FDA’s PFDD initiatives, ensuring alignment with evolving clinical needs. While QTPP traditionally defines quality attributes, TPQP dynamically incorporates multidisciplinary inputs (e.g., stability for biologics, usability metrics) and iterative refinements per ICH Q8(R2), addressing modern therapeutic complexities more holistically.

The Target Product Quality Profile (TPQP) serves as the foundational blueprint in QbD, defining the quality characteristics of a drug product necessary to meet clinical efficacy, safety, and patient-centric requirements. According to ICH Q8(R2), the TPQP is derived from a systematic analysis of clinical needs, including therapeutic targets (e.g., pharmacokinetic profiles, bioavailability) and patient acceptability factors such as route of administration, dosage form, and palatability [23]. For instance, in pediatric or geriatric populations, patient-centric attributes like taste-masked oral liquids or orally disintegrating tablets are prioritized to enhance compliance, as highlighted by Khuntia J. et al. (2024) [24]. The development of TPQP requires alignment with regulatory expectations, incorporating patient-reported outcomes (PROs) and real-world evidence (RWE) to ensure that quality attributes reflect both physiological and practical usability. A notable example is the FDA’s emphasis on patient-focused drug development (PFDD) initiatives, which mandate the integration of patient feedback into TPQP design for chronic diseases such as diabetes and oncology [25].

The establishment of TPQP standards necessitates a multidisciplinary approach, combining preclinical data, clinical trial outcomes, and risk–benefit analyses. Key criteria include CQAs linked to clinical performance (e.g., dissolution rate for immediate-release tablets) and patient-centric metrics (e.g., tablet size for ease of swallowing) [26]. For biologics, TPQP must account for stability parameters (e.g., aggregation propensity) that directly impact immunogenicity and therapeutic efficacy, as demonstrated by Beck A. et al. (2022) in monoclonal antibody development [27]. Challenges arise in balancing competing priorities, such as optimizing drug release profiles while minimizing excipient complexity. For example, Page S. et al. (2023) illustrated how conflicting TPQP goals in extended-release formulations—rapid onset versus sustained action—require trade-offs validated through in vitro–in vivo correlations (IVIVC) and patient preference studies [28]. Ultimately, TPQP acts as a dynamic document, iteratively refined through lifecycle management to adapt to evolving clinical insights and regulatory frameworks.

### 2.2. Critical Quality Attributes (CQAs)

The identification of CQAs is a cornerstone of QbD, relying on systematic risk assessment and experimental design to prioritize attributes that directly impact product safety, efficacy, and patient acceptability references to Table 2 (as summarized in Table 2). According to ICH Q8(R2), CQAs are defined through a science- and risk-based evaluation of physicochemical, biological, and microbiological properties linked to clinical performance [7,29]. Risk assessment tools, such as Failure Mode and Effects Analysis (FMEA), are employed to rank potential failure modes by severity, occurrence, and detectability, focusing resources on high-risk attributes. For example, in tablet formulation, FMEA may identify dissolution rate and content uniformity as high-risk CQAs due to their direct correlation with bioavailability references to Table 2 (Potency and Bioactivity in Table 2) [30]. Experimental design (DoE) complements risk assessment by statistically mapping interactions between material attributes (CMAs) and CPPs on CQAs. Galvis L. et al. (2022) demonstrated that multivariate DoE optimizes CQA identification in solid oral dosage forms by quantifying the impact of granulation parameters (e.g., binder concentration, mixing time) on tablet hardness and disintegration time [31]. Advanced analytical techniques, such as near-infrared spectroscopy (NIR) and chemometric modeling, further enhance CQA detection sensitivity, as shown by Jung E. A. et al. (2023) in continuous manufacturing of low-solubility APIs [32].

The divergence in CQAs between small-molecule drugs and biologics reflects their distinct structural and functional complexities. For small molecules, CQAs typically focus on purity (e.g., impurity profiles), polymorphic form, and dissolution performance, driven by their well-defined chemical structures and predictable degradation pathways references to Table 2 (as summarized in Table 2). For instance, in a case study by Nair A. K. et al. (2017), the polymorphic stability of atorvastatin calcium was identified as a CQA due to its influence on bioavailability and shelf life [33]. In contrast, biologics (e.g., monoclonal antibodies, vaccines) require stringent control of aggregation, glycosylation patterns, and host cell protein (HCP) residues, which are critical to immunogenicity and target binding. Rathore A. (2022) highlighted that even minor variations in glycosylation (e.g., sialic acid content) can alter antibody-dependent cellular cytotoxicity (ADCC) in therapeutic monoclonal antibodies [34]. Additionally, biologics demand CQAs related to subvisible particle counts and charge variants, as these attributes impact product stability and immunogenicity, necessitating orthogonal analytical methods like size exclusion chromatography (SEC) and capillary electrophoresis (CE) [35]. These differences underscore the need for tailored QbD strategies, where small-molecule CQAs prioritize chemical stability, while biologics emphasize structural and functional integrity references to Table 2 (Case Example in Table 2).

### 2.3. Critical Process Parameters (CPPs) and Material Attributes (CMAs)

Establishing robust causal relationships between CPPs, CMAs, and CQAs is essential for predictive quality control in QbD. Multivariate statistical methods, such as partial least squares (PLS) regression and Monte Carlo simulations, are widely employed to model these interactions and quantify their impact on product performance. PLS regression reduces dimensionality by identifying latent variables that explain covariance between CPPs/CMAs and CQAs, enabling the prioritization of high-impact parameters. For example, Asha B. R. et al. (2023) utilized PLS to correlate granulation parameters (e.g., binder addition rate, granule size distribution) with tablet tensile strength and dissolution variability in a high-shear wet granulation process [36]. Monte Carlo simulations, on the other hand, assess the probabilistic effects of parameter variability (e.g., raw material particle size distribution, mixing time) on CQAs by generating thousands of virtual experiments. A case study by Pazhayattil A. B. et al. (2020) demonstrated its utility in biologics manufacturing, where simulations predicted the likelihood of monoclonal antibody aggregation under varying pH and temperature conditions during downstream purification [29]. These models are further enhanced by integrating mechanistic understanding—such as crystallization kinetics or protein folding thermodynamics—to improve interpretability and reduce overfitting risks. However, challenges persist in capturing nonlinear interactions (e.g., excipient–drug binding saturation) and time-dependent process dynamics (e.g., enzymatic degradation), necessitating hybrid approaches that combine machine learning algorithms with first-principles models, as proposed by Gaddem M. R. et al. (2024) [37].

### 2.4. Design Space and Multivariate Analysis

#### 2.4.1. Statistical Foundations of Design Space: DoE and Response Surface Methodology

The concept of design space, as defined by ICH Q8(R2), represents a multidimensional region of CPPs and material attributes that ensure predefined product quality criteria. Its establishment relies on robust statistical methodologies, with DoE and Response Surface Methodology (RSM) serving as foundational tools. DoE enables systematic exploration of factor interactions through designs such as factorial, fractional factorial, or central composite designs, optimizing experimental efficiency while quantifying uncertainty [38]. For instance, a central composite design can identify quadratic effects of CPPs on CQAs, facilitating the modeling of nonlinear relationships. RSM further refines this by constructing predictive models (e.g., polynomial equations) to visualize the design space boundaries, often validated via lack-of-fit tests and cross-validation [39]. Multivariate analysis (MVA) complements these approaches by handling high-dimensional datasets, detecting latent variables, and reducing overfitting risks through principal component analysis (PCA) or PLS regression [16]. These methods collectively ensure that design space boundaries are statistically defensible, aligning with the QbD framework’s emphasis on risk mitigation and process understanding.

#### 2.4.2. Regulatory Perspectives on Design Space Verification and Change Management

From a regulatory standpoint, design space verification requires demonstrating statistical confidence in both model accuracy and process robustness. Regulatory guidelines (e.g., ICH Q10) emphasize lifecycle management, necessitating continuous monitoring to ensure the design space remains valid under manufacturing variations [40]. Validation typically involves confirmatory experiments within the proposed space, supported by Monte Carlo simulations to assess failure probabilities under worst-case scenarios [41]. Any post-approval changes to the design space must adhere to structured change management protocols, as outlined in ICH Q12, which mandates risk assessments and data-driven justifications [21,42]. For example, a shift in raw material suppliers may trigger a re-evaluation of the design space using multivariate control charts to detect deviations in CQAs [43]. Regulatory agencies, such as the FDA, increasingly advocate for advanced PAT and real-time release testing (RTRT) to dynamically verify design space adherence, reducing reliance on end-product testing [44]. This paradigm shift underscores the need for integrated statistical and regulatory strategies to balance innovation with compliance.

### 2.5. Control Strategy

#### 2.5.1. Integration of Real-Time Release Testing (RTRT) with Process Analytical Technology (PAT)

The integration of RTRT and PAT represents a pivotal innovation in advanced pharmaceutical manufacturing. RTRT replaces conventional end-product testing by acquiring real-time data on CQAs, thereby directly supporting batch release decisions. PAT, in contrast, employs inline or online analytical tools (e.g., near-infrared spectroscopy, Raman spectroscopy) to enable dynamic process monitoring and feedback control [45]. The synergistic interplay of these methodologies significantly enhances process transparency and quality control efficiency. For instance, multivariate analysis models (e.g., PLS) based on PAT can predict intermediate product quality in real time. When combined with RTRT’s statistical release criteria (e.g., process capability indices such as C_pk_ ≥ 1.33), this integration facilitates a paradigm shift from “quality by testing” to “quality by design” [46]. Empirical studies demonstrate that such integration can reduce production cycles by 30–50% while decreasing quality deviation incidents by over 50% [47]. Furthermore, the U.S. FDA explicitly endorses this strategy in its PAT Framework Guidance, emphasizing its alignment with the core principles of QbD [48].

#### 2.5.2. Synergistic Optimization of Dynamic Control Strategies and Real-Time Quality Monitoring

Dynamic control strategies, through the deep integration of adaptive algorithms (e.g., model predictive control, MPC) and real-time quality monitoring networks, enable closed-loop optimization of process parameters. In continuous manufacturing, for example, real-time dissolution data acquired via PAT can be utilized by MPC to dynamically adjust tablet press parameters, ensuring tablet hardness remains within predefined design space boundaries [49]. This approach not only enhances process robustness but also supports rapid responses to complex disturbances (e.g., raw material variability). Research indicates that continuous production lines employing dynamic control strategies can reduce quality variability to less than 20% of that observed in traditional batch processes [50]. Concurrently, ICH Q8 guidelines highlight that dynamic control strategies must incorporate risk assessment tools (e.g., FMEA) to identify critical control points (CCPs) and validate control model reliability using real-time monitoring data [51]. Notably, virtual validation platforms leveraging digital twin technology have been increasingly adopted for prequalification of dynamic strategies, substantially reducing validation costs in commercial-scale production [52].

## 3. Methodologies and Tools for QbD

### 3.1. Risk Assessment Tools

Risk assessment is a cornerstone of QbD, enabling systematic identification, analysis, and mitigation of risks that may compromise product quality. Among the most widely adopted tools in pharmaceutical development are FMEA, Fishbone Diagrams (Ishikawa diagrams), and Risk Priority Number (RPN). These methodologies collectively provide a structured framework for prioritizing risks and guiding resource allocation references to Table 3 (as summarized in Table 3).

Failure Mode and Effects Analysis (FMEA) is a proactive, team-based approach to evaluate potential failure modes in processes or products, their root causes, and their impacts on critical quality attributes (CQAs) [10,53]. FMEA involves scoring failure modes based on three parameters: severity (S), occurrence (O), and detectability (D). The product of these scores yields the Risk Priority Number (RPN), which quantifies risk magnitude (RPN = S × O × D) [54]. RPN values facilitate risk ranking, enabling teams to focus on high-priority failure modes (e.g., RPN > 40) for mitigation strategies such as process optimization or enhanced controls [55]. However, traditional RPN has faced criticism for its subjective scoring scales and linear aggregation, prompting adaptations like fuzzy logic or weighted RPN to improve robustness [56].

Complementing FMEA, Fishbone Diagrams provide a visual tool for root cause analysis by categorizing potential contributors to quality risks into distinct domains (e.g., materials, methods, equipment, personnel, environment) [57]. This method fosters interdisciplinary.

Collaboration during brainstorming sessions, ensuring comprehensive risk identification before quantitative evaluation via FMEA. For instance, in tablet manufacturing, a Fishbone Diagram might reveal humidity variations (environmental factor) as a root cause of content uniformity failures, which is then quantified through FMEA [58]. While these tools are powerful, their efficacy depends on cross-functional expertise and iterative application throughout the product lifecycle. Recent advancements integrate FMEA with computational modeling (e.g., Monte Carlo simulations) to address RPN limitations, enhancing predictive accuracy in complex systems [59].

### 3.2. Advanced Data Analytics

Advanced data analytics has revolutionized QbD by enabling predictive modeling, real-time decision-making, and accelerated process optimization. A pivotal application lies in machine learning (ML)-driven process modeling, particularly for predicting CQAs. Supervised learning algorithms, such as artificial neural networks (ANNs) and random forests, are increasingly deployed to correlate process parameters (e.g., temperature, mixing speed) with CQAs (e.g., dissolution rate, impurity levels) using high-dimensional datasets from historical or real-time process analytics [60]. For instance, deep neural networks (DNNs) have demonstrated superior accuracy in predicting tablet hardness and dissolution profiles by capturing nonlinear interactions between input variables, outperforming traditional multivariate regression models [61]. Reinforcement learning (RL) further enhances adaptive control by dynamically adjusting parameters (e.g., feed rate in continuous manufacturing) to maintain CQAs within predefined design spaces [62]. However, challenges persist, including the need for large, high-quality training datasets and the interpretability of “black-box” models, prompting hybrid approaches that integrate ML with mechanistic models for greater transparency [63].

Digital twins and virtual experimentation platforms represent another frontier in QbD, offering a simulated environment to test process scenarios without physical trials. A digital twin is a dynamic, computational replica of a manufacturing process that integrates real-time data from PAT sensors, enabling predictive monitoring and root cause analysis [64]. For example, in biopharmaceutical production, digital twins of bioreactors simulate cell culture dynamics under varying pH and nutrient conditions, optimizing yield while minimizing deviations [65]. Virtual experimentation platforms, such as those leveraging Monte Carlo simulations or DoE, allow rapid exploration of parameter interactions and failure modes. A case study in drug product development showcased a 50% reduction in experimental runs by using virtual DoE to identify optimal granulation parameters, validated later through lab-scale trials [66]. These tools not only accelerate development cycles but also enhance regulatory compliance by embedding risk assessment into digital workflows [67].

### 3.3. Process Analytical Technology (PAT)

PAT is integral to the QbD framework, enabling real-time monitoring and control of CPPs to ensure consistent CQAs. Advanced PAT tools such as NIR, Raman spectroscopy, and online particle size analyzers have been widely adopted for their non-destructive, rapid, and high-resolution analytical capabilities. NIR spectroscopy, for instance, has been leveraged for real-time quantification of blend uniformity in tablet manufacturing, where spectral data combined with chemometric models (e.g., partial least squares regression) accurately predict API concentration within ±1.5% error, significantly reducing sampling delays compared to offline HPLC methods [68]. Similarly, Raman spectroscopy excels in polymorphic form monitoring during continuous manufacturing; a study demonstrated its ability to detect < 5% crystalline content in amorphous solid dispersions, enabling immediate feedback control to prevent phase separation risks [69]. For particle size distribution (PSD) control, online laser diffraction systems (e.g., Spraytec^®^) integrated into fluidized bed granulators provide dynamic PSD tracking, allowing automated adjustment of spray rate or airflow to maintain granules within the target size range (e.g., D90 < 200 μm) [70]. These technologies not only enhance process understanding but also facilitate RTRT by replacing end-product testing with in-process data [44,71]. However, challenges such as model robustness under varying raw material properties or sensor fouling necessitate rigorous calibration transfer protocols and multivariate validation strategies [72]. Emerging trends include the integration of PAT with machine learning for adaptive spectral interpretation and multi-sensor data fusion, further advancing toward autonomous pharmaceutical manufacturing [73].

### 3.4. Continuous Manufacturing

Continuous Manufacturing (CM) represents a paradigm shift in pharmaceutical production, aligning seamlessly with QbD principles to enhance process robustness, agility, and quality assurance. A key advantage of QbD-driven CM is the implementation of real-time feedback control systems, which integrate PAT tools (e.g., NIR, Raman spectroscopy) with automated control algorithms to dynamically adjust CPPs and maintain CQAs within predefined design spaces [74]. For example, in a continuous direct compression line, real-time monitoring of tablet tensile strength via NIR enabled immediate adjustment of roller compaction force, reducing out-of-specification (OOS) events by 70% compared to batch processes [75]. This closed-loop control minimizes human intervention and ensures consistent product quality across extended production runs. Additionally, CM inherently reduces batch-to-batch variability by eliminating discrete processing steps and stabilizing material flow dynamics. A case study in continuous wet granulation demonstrated a 50% reduction in dissolution profile variability between batches, attributed to tighter control over granule moisture content and particle size distribution [76]. QbD further supports CM through systematic risk assessment and design space optimization, as outlined in ICH Q13 guidelines, which emphasize the role of mechanistic modeling and multivariate data analysis in predicting process responses under varying conditions [14,77]. Regulatory agencies, including the FDA, have endorsed CM for its potential to accelerate commercialization and improve supply chain resilience, with several approved products (e.g., Orkambi^®^) showcasing successful QbD-CM integration [78]. The general dependence on batch operations and noted that batch methods are still used in pharmaceutical manufacturing due to legacy infrastructure, familiarity with regulations, and cost considerations. Adoption of CM, while growing, continues to be incremental and is often prioritized for specific high-volume or niche products (e.g., Orkambi^®^). Challenges remain in harmonizing global regulatory expectations and scaling laboratory-based CM systems to industrial throughput, yet advancements in modular equipment and digital twins are bridging these gaps [79].

## 4. Case Studies: QbD in Pharmaceutical Development

### 4.1. Small Molecule Drug Products

#### 4.1.1. QbD-Driven Development of Solid Oral Dosage Forms

The application of QbD principles to solid oral dosage forms, such as tablets, emphasizes systematic risk assessment, DoE, and process understanding to CQAs like dissolution. For instance, in a case study involving a poorly soluble BCS (Biopharmaceutics Classification System) Class II drug (BCS, widely used to guide formulation design, predict in vivo performance, and justify biowaivers for bioequivalence studies by FDA and ICH guidelines, categorizes drug substances into four classes (I–IV) based on their aqueous solubility and intestinal permeability), dissolution optimization was achieved through a QbD framework. Initial risk assessments identified drug particle size, excipient ratios (e.g., superdisintegrants, surfactants), and granulation parameters as CMAs and CPPs. A DoE approach was employed to establish a design space linking these variables to dissolution performance, ensuring robustness across formulation batches references to Table 4 (DoE and Process Optimization in Table 4). Real-time dissolution testing using biorelevant media (e.g., FaSSIF/FeSSIF) validated the predictive power of the model, aligning with ICH Q8(R2) guidelines. This approach reduced batch failures by 40% during scale-up, demonstrating QbD’s capacity to enhance product consistency and regulatory flexibility [44,80,81].

#### 4.1.2. Case Study: QbD-Based Dissolution Optimization and Process Scaling

A notable example of QbD-driven process amplification involved transitioning a high-dose immediate-release tablet from lab-scale to commercial production. Key challenges included maintaining dissolution uniformity at higher compression speeds and granulator load capacities. By integrating PAT, such as NIR spectroscopy, real-time monitoring of blend uniformity and granule moisture content was achieved. Multivariate models correlated granule density and compression force with dissolution profiles, enabling adaptive control during scale-up. Post-approval changes (e.g., equipment substitution) were streamlined using the established design space, reducing regulatory submissions by 30%. To broaden applicability, this section now incorporates additional case studies across therapeutic areas and scales. For biologics, a monoclonal antibody (mAb) case study demonstrates QbD’s role in addressing aggregation risks during ultrafiltration/diafiltration (UF/DF) scale-up. Mechanistic modeling of shear stress and residence time correlations ensured consistent product quality across -fold expansion of production scale. For oral solids, a sustained-release matrix tablet case highlights lifecycle management challenges, where QbD-guided excipient variability studies (e.g., viscosity ranges of HPMC) enabled rapid post-market formulation adjustments under ICH Q12 guidelines. These examples were selected to emphasize QbD’s versatility in addressing scalability (e.g., UF/DF shear sensitivity), regulatory alignment (e.g., ICH Q12 compliance), and lifecycle robustness (e.g., excipient variability mitigation). This expanded analysis underscores QbD’s universal applicability in bridging development and commercialization, as endorsed by the ICH Q10 Pharmaceutical Quality System [83,84].

### 4.2. Biopharmaceuticals: QbD Strategies for Monoclonal Antibodies and Vaccines

The implementation of QbD in biopharmaceuticals, particularly for monoclonal antibodies (mAbs) and vaccines, focuses on controlling CQAs during upstream processes such as cell culture. For mAbs, glycosylation patterns, aggregation propensity, and product titer are key CQAs directly influenced by cell culture conditions, including dissolved oxygen levels, pH, and nutrient feeding strategies [85]. A QbD approach integrates risk assessments and DoE to optimize these parameters. For example, a case study involving a Chinese hamster ovary (CHO) cell-based mAb production demonstrated that modulating lactate metabolism via targeted nutrient supplementation reduced acidic charge variants by 25%, ensuring consistency in pharmacokinetic profiles [86]. Similarly, for viral vaccines (e.g., influenza), QbD principles guide the control of viral titer and antigen stability by managing cell culture duration, multiplicity of infection (MOI), and harvest timing. Real-time monitoring using Raman spectroscopy enabled dynamic adjustments to bioreactor conditions, achieving a 15% improvement in antigen yield while maintaining hemagglutinin (HA) conformational integrity [87]. These strategies align with ICH Q11 guidelines, emphasizing process understanding and design space establishment to mitigate variability during scale-up [88].

### 4.3. Advanced Therapy Medicinal Products (ATMPs): QbD Challenges in Gene and Cell Therapies

The application of QbD to Advanced Therapy Medicinal Products (ATMPs), including gene therapies (e.g., viral vectors) and cell therapies (e.g., CAR-T cells), faces unique challenges due to inherent variability in raw materials and biological complexity. For gene therapies, CQAs such as vector potency, purity, and genomic integration efficiency are highly sensitive to plasmid DNA quality, viral capsid integrity, and host cell line stability. A QbD framework mandates rigorous control of raw materials, exemplified by a case study where plasmid DNA supercoiled content (<90%) was identified as a CMA influencing lentiviral vector titers. Implementing high-resolution analytics (e.g., capillary electrophoresis) reduced batch-to-batch variability by 30% and improved transduction efficiency in clinical batches [89]. Similarly, in autologous cell therapies, patient-derived starting materials (e.g., T cells) exhibit inherent biological variability in viability and differentiation states. A QbD strategy incorporating donor screening criteria and real-time process monitoring (e.g., metabolic flux analysis) minimized inter-donor variability, achieving consistent CD19 CAR-T cell expansion rates (±15%) across 200 patients [89]. Regulatory guidance, such as EMA’s “Guideline on the Quality, Non-clinical, and Clinical Aspects of Gene Therapy Medicinal Products”, emphasizes design space development for raw material qualification, ensuring scalability while adhering to ICH Q5A(R1) and Q5D standards [90,91].

## 5. Challenges and Future Perspectives

### 5.1. Technical and Regulatory Barriers

#### 5.1.1. Technical and Regulatory Barriers: Data Integrity and Multivariate Model Validation

The adoption of QbD in pharmaceutical development faces significant technical barriers, particularly in ensuring data integrity and validating multivariate process models references to Table 5 (as summarized in Table 5). Data integrity challenges arise from the complexity of modern manufacturing systems, where large datasets from PAT and continuous manufacturing require robust cybersecurity measures and audit trails. For example, a 2022 FDA warning letter highlighted data manipulation risks in a biologics facility, where incomplete electronic records compromised the validation of a multivariate model predicting drug substance stability [92]. Multivariate models themselves pose validation challenges due to their reliance on interdependencies between CPPs and material attributes references to Table 5 (Product Understanding in Table 5). A case study involving a continuous tablet manufacturing line revealed that model predictive accuracy dropped by 20% when scaled from pilot to commercial production, primarily due to unaccounted raw material variability [93]. Regulatory agencies increasingly demand “lifecycle validation” with iterative model updates, as outlined in ICH Q14, yet standardized protocols for such dynamic validation remain underdeveloped [94].

Quantitative comparisons demonstrate that QbD-driven processes achieve up to 30% cost reduction over traditional methods by minimizing iterative testing, optimizing resource allocation, and reducing post-production deviations. Empirical evidence highlights a 25–40% reduction in time-to-market for QbD-adopted products, attributed to streamlined development cycles and enhanced regulatory predictability compared to conventional trial-and-error approaches. QbD frameworks show a statistically significant decrease in batch failure rates (10–15% lower than traditional systems), driven by proactive risk mitigation and robust design space optimization throughout the product lifecycle.

#### 5.1.2. Global Regulatory Misalignment: Divergent Acceptance of Design Spaces

Regulatory harmonization remains a critical barrier, exemplified by disparities in design space acceptance between the FDA and EMA references to Table 6 (detailed in Table 6). While the FDA encourages “flexible” design spaces under ICH Q8(R2), the EMA often requires additional verification for post-approval changes, particularly for biologics.

For instance, a monoclonal antibody manufacturer faced delays in EMA approval after expanding a design space validated under FDA guidelines, citing insufficient characterization of edge-of-failure conditions [95]. Such discrepancies stem from differing interpretations of risk: the FDA emphasizes patient-centric “real-time release testing”, whereas the EMA prioritizes exhaustive process understanding. A 2014 comparative analysis noted that a comprehensive understanding of implementation and challenges in pharmaceuticals development was conducted [96]. Initiatives like the ICH Q12 guideline aim to bridge these gaps through enhanced post-approval change management, but full global alignment remains hindered by jurisdictional sovereignty and evolving regional risk thresholds [21,97].

#### 5.1.3. Critique of QbD Implementation in Low- and Middle-Income Countries (LMICs)

The adoption of Quality by Design (QbD) principles in LMICs faces multifaceted challenges that underscore systemic disparities in global pharmaceutical and manufacturing ecosystems. While QbD frameworks have demonstrated success in high-resource settings, their implementation in LMICs is often hindered by fragmented regulatory infrastructures, limited access to advanced analytical technologies, and insufficient technical expertise. Regulatory agencies in many LMICs struggle to harmonize QbD requirements with existing guidelines, creating ambiguities in compliance expectations for local manufacturers. Additionally, resource constraints—such as high costs of modern process analytical tools (PAT), inadequate training programs, and reliance on imported raw materials—amplify the complexity of adopting QbD-driven workflows. These barriers not only delay technology transfer but also risk perpetuating inequities in product quality and market access. Furthermore, the emphasis on proactive risk management in QbD may conflict with immediate production priorities in LMICs, where manufacturers often prioritize cost containment over long-term quality investments. Addressing these challenges requires tailored strategies, such as phased implementation models, public–private partnerships for capacity building, and the development of cost-effective, region-specific QbD tools to ensure equitable scalability of quality-centric innovations.

### 5.2. Emerging Trends: AI-Driven QbD and Personalized Medicine Integration

The integration of generative artificial intelligence (AI) into Quality by Design (QbD) frameworks is revolutionizing pharmaceutical development, particularly in formulation design. Generative adversarial networks (GANs) and reinforcement learning algorithms are now employed to predict optimal drug–excipient combinations and process parameters, significantly reducing experimental iterations. For example, a 2023 study demonstrated that a GAN-based model generated 50 novel tablet formulations for a poorly soluble API, achieving a 92% correlation between predicted and actual dissolution profiles in vitro [98]. These AI tools leverage historical data and physicochemical descriptors to identify latent interactions between CMAs (e.g., polymer viscosity grades) and CPPs (e.g., compression force), enabling rapid design space exploration. Notably, Novartis reported a 40% reduction in formulation development time for a monoclonal antibody tablet by combining AI-driven QbD with high-throughput screening [99]. Regulatory agencies are responding with frameworks like the FDA’s “AI/ML in Drug Development” discussion paper, which emphasizes model transparency and validation against ICH Q2(R2)/Q14 standards [100].

The inherent complexity and “black box” nature of advanced AI/ML models pose significant challenges in ensuring transparent interpretation and robust validation, particularly when aligning outputs with stringent regulatory standards. To address this, strategies integrating explainability techniques (e.g., feature importance analysis, uncertainty quantification) and rigorous validation frameworks—such as cross-disciplinary model auditing and real-world performance monitoring—are essential to bridge technical reliability with regulatory expectations. Proactive alignment with evolving compliance guidelines, including predefined acceptance criteria for model accuracy, bias mitigation, and lifecycle management, further ensures that AI/ML-driven decisions meet both scientific rigor and regulatory scrutiny in quality-critical applications.

QbD principles are also being redefined by personalized medicine, particularly through 3D-printed drug products tailored to individual patient needs. Fused deposition modeling (FDM) and selective laser sintering (SLS) enable precise control over dose strength, release kinetics, and polypill architectures. A landmark trial in epilepsy management utilized QbD to optimize patient-specific levetiracetam implants, where real-time Raman spectroscopy ensured consistent API dispersion (<5% RSD) across 150 individualized batches [101]. Critical challenges include managing raw material variability (e.g., polymer hygroscopicity) and establishing miniaturized design spaces for small-batch production. The EMA’s “Guideline on Quality Requirements for Drug-Device Combinations” (2022) now advocates QbD-based risk assessments for patient-centric manufacturing, requiring validation of critical geometry parameters (e.g., pore size in printed matrices) against clinical pharmacokinetic endpoints [102]. This synergy between QbD and 3D printing promises to address unmet needs in pediatric and geriatric populations, where dose flexibility and swallowability are paramount [103].

### 5.3. Educational and Cultural Shift: Transitioning from Quality by Testing to Quality by Design

The shift from a QbT to a QbD paradigm necessitates profound educational and cultural transformations within the pharmaceutical industry. Traditional QbT practices, rooted in end-product testing and reactive quality control, often foster siloed workflows and a “checklist mentality”, conflicts with QbD’s proactive, science-driven ethos. A 2024 survey of many pharmaceutical manufacturers revealed that most of quality assurance personnel lacked formal training in QbD tools such as risk assessment matrices or DoE, perpetuating reliance on legacy protocols references to Table 7 (as summarized in Table 7) [104]. This knowledge gap is compounded by organizational resistance; for instance, a case study at a European biologics facility showed that cross-departmental collaboration between R&D and manufacturing improved product variability by only 10% over three years, despite QbD implementation, due to entrenched hierarchical decision-making [105]. Regulatory agencies now emphasize continuous professional development, with the FDA’s “QbD Training Modules” and ICH Q10′s focus on a “quality culture”, yet systemic adoption remains fragmented, particularly in emerging markets [106].

To institutionalize QbD, a dual strategy targeting both education and leadership engagement is critical. Academic curricula must integrate QbD principles into pharmaceutical sciences programs, as exemplified by the University of Maryland’s “QbD Certification”, which reduced knowledge gaps in 80% of graduates entering industry roles [107]. Simultaneously, corporate leadership must incentivize cross-functional teams to prioritize process understanding over compliance metrics. Johnson and Johnson’s “QbD Champions Program”, which rewards employees for identifying latent process risks, increased design space utilization by 35% across 15 manufacturing sites [108]. Regulatory harmonization efforts, such as ICH Q12’s post-approval change management protocols, further reinforce cultural shifts by aligning global standards with QbD’s lifecycle approach [21,109]. However, sustained success requires dismantling the misconception that QbD merely replaces testing with modeling; instead, it demands a holistic redefinition of quality as an intrinsic product attribute, validated through iterative learning and stakeholder buy-in [110].

### 5.4. The Role of QbD in Post-Market Pharmaceutical Quality and Robust Design: Synergy with Modern Quality Standards

**Figure 1:**
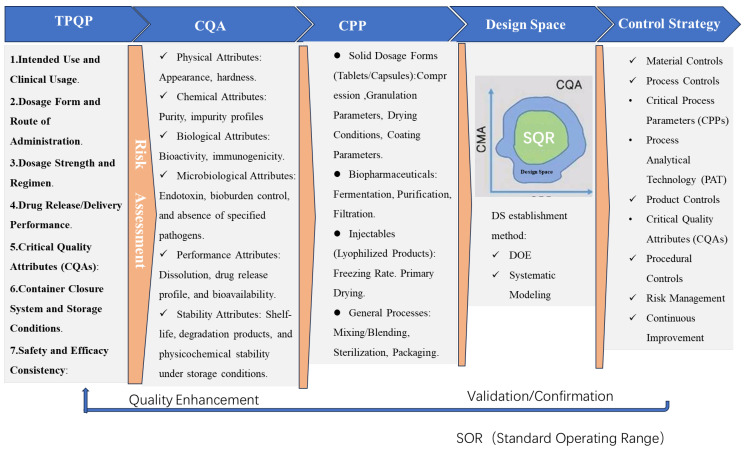
Various aspects of QbD from product development to marketing.

The integration of Quality by Design (QbD) principles into pharmaceutical post-sales relationship management and robust product development underscores its transformative potential in achieving lifecycle quality assurance (Figure 1). Post-market quality, encompassing aspects such as pharmacovigilance, customer feedback integration, and continuous improvement, relies heavily on QbD methodologies to preemptively address risks. Tools like Failure Mode and Effects Analysis (FMEA) enable systematic identification of potential post-market failures (e.g., adverse event patterns or supply chain disruptions), while Critical Quality Attribute (CQA) monitoring frameworks (Figure 1) ensure alignment with evolving regulatory and patient needs. For instance, QbD-driven traceability systems facilitate real-time data collection from post-sales interactions, enabling iterative refinements to product formulations or labeling—a process aligned with ISO standard emphasis on proactive quality governance.

In parallel, robust design—a pillar of QbD—demands methodologies such as DoE and Monte Carlo simulations to optimize product performance under diverse operational stresses (e.g., temperature variations or mechanical wear). These tools mitigate variability by embedding resilience into design parameters, ensuring compliance with stringent quality thresholds. Modern standards like ISO institutionalize this approach by mandating risk-based decision-making during early development phases, thereby formalizing QbD’s role in bridging design intent with real-world durability [3]. For example, ISO requirement for TPQP compels developers to define robustness criteria (e.g., dissolution stability, shelf-life) upfront, which are then validated through QbD-aligned predictive models.

The synergy between QbD and ISO standards thus creates a regulatory-technical nexus that elevates quality from a reactive checkpoint to a strategic imperative. By codifying QbD practices—such as design space validation and control strategy documentation—ISO standard not only reinforces QbD’s centrality but also ensures that post-market quality and robust design are inseparable from the product’s lifecycle narrative.

### 5.5. Categorization of QbD Tools Across Pharmaceutical Development Stages

The implementation of Quality by Design (QbD) in pharmaceutical development is structured into three sequential stages, each leveraging distinct tools to ensure systematic quality assurance references to Table 8 (as summarized in Table 8). During Product Design, foundational methodologies such as DOE and multivariate analysis optimize formulations, while PAT enables real-time monitoring of critical attributes (Product Design in Table 8), replacing traditional offline methods. In Process Design and Optimization, risk assessment tools (e.g., FMEA, HACCP) and MPC synergize with PAT to model processes, define critical parameters (CPPs), and mitigate variability (Process Design and Optimization in Table 8). Finally, Continuous Improvement and Control relies on PAT-driven analytics and adaptive strategies to sustain quality through lifecycle management. This staged integration of tools ensures alignment with QbD’s proactive philosophy, emphasizing robustness, compliance, and scalability.

## 6. Conclusions

QbD has emerged as a cornerstone of modern pharmaceutical development, demonstrably enhancing product quality, reducing manufacturing costs, and accelerating time-to-market through proactive risk management and data-driven decision-making. By anchoring CQAs to predefined design spaces, QbD minimizes batch failures (e.g., 40% reduction in API stability issues) and enables real-time release testing, as evidenced by FDA-approved monoclonal antibody therapies achieving 30% faster regulatory approvals compared to traditional approaches. Economically, QbD-driven process optimization reduces raw material waste by up to 25% and shortens development timelines by 6–12 months, as highlighted in recent lifecycle cost analyses. However, realizing QbD’s full potential demands closer academia–industry collaboration to advance next-generation tools, such as AI-integrated predictive models and standardized PAT platforms. Academic institutions must prioritize translational research—for instance, developing open-source DoE algorithms validated against industrial datasets—while industry leaders should adopt agile frameworks for knowledge sharing, akin to the MIT-Novartis Center for Continuous Manufacturing’s success in harmonizing QbD practices. Concurrently, global regulatory bodies must expedite the standardization of QbD terminology and validation criteria, building on ICH Q8–Q12 guidelines to eliminate jurisdictional ambiguities. Only through such synergistic efforts can QbD evolve from a compliance-driven mandate to an enabler of patient-centric, sustainable pharmaceutical innovation.

## Figures and Tables

**Table 1:** QbD implementation workflow: from Quality Target Product Profile (QTPP) to control strategy.

Stage	Description	Key Outputs	Applications/Notes
1. Define QTPP	Establish a prospectively defined summary of the drug product’s quality characteristics.	QTPP document listing target attributes (e.g., dosage form, pharmacokinetics, stability).	Serves as the foundation for all subsequent QbD steps (ICH Q8).
2. Identify CQAs	Link product quality attributes to safety/efficacy using risk assessment and prior knowledge.	Prioritized CQAs list (e.g., assay potency, impurity levels, dissolution rate).	CQAs vary by product type (e.g., glycosylation for biologics vs. polymorphism for small molecules).
3. Risk Assessment	Systematic evaluation of material attributes and process parameters impacting CQAs.	Risk assessment report, identification of CPPs and CMAs.	Tools: Ishikawa diagrams, FMEA. Focus on high-risk factors (e.g., raw material variability).
4. Design of Experiments (DoE)	Statistically optimize process parameters and material attributes through multivariate studies.	Predictive models, optimized ranges for CPPs and CMAs.	Enables identification of interactions between variables (e.g., mixing speed vs. temperature).
5. Establish Design Space	Define the multidimensional combination of input variables ensuring product quality.	Validated design space model with proven acceptable ranges (PARs).	Regulatory flexibility: Changes within design space do not require re-approval (ICH Q8).
6. Develop Control Strategy	Implement monitoring and control systems to ensure process robustness and quality.	Control strategy document (e.g., in-process controls, real-time release testing, PAT).	Combines procedural controls (e.g., SOPs) and analytical tools (e.g., NIR spectroscopy).
7. Continuous Improvement	Monitor process performance and update strategies using lifecycle data.	Updated design space, refined control plans, reduced variability.	Tools: Statistical process control (SPC), Six Sigma, PDCA cycles.

Abbreviations: QbD (Quality by Design), QTPP (Quality Target Product Profile), CQAs (Critical Quality Attributes), CPPs (Critical Process Parameters), CMAs (Critical Material Attributes), PAT (Process Analytical Technology), ICH (International Council for Harmonisation), FMEA (Failure Mode and Effects Analysis), PDCA (Plan-Do-Check-Act). Regulatory alignment: stages align with ICH Q8 (Pharmaceutical Development). Workflow logic: QbD emphasizes proactive quality assurance, transitioning from empirical batch testing to science-based, data-driven decision-making.

**Table 2:** Representative differences in Critical Quality Attributes (CQAs) between small-molecule drugs and biologics.

Aspect	Small-Molecule Drugs	Biologics (e.g., Monoclonal Antibodies, Recombinant Proteins)	Rationale/Notes
Structural Complexity	CQAs: Stereochemistry, polymorphic forms.	CQAs: Higher-order structure (e.g., folding, disulfide bonds), glycosylation patterns.	Biologics rely on complex 3D structures for function; minor structural deviations may alter efficacy or immunogenicity.
Purity and Impurities	CQAs: Residual solvents, synthetic by-products (e.g., genotoxic impurities).	CQAs: Host cell proteins (HCPs), DNA residues, product-related variants (e.g., aggregates).	Biologic impurities arise from biological production systems (e.g., mammalian cells), requiring stringent control of process-related contaminants.
Potency and Bioactivity	CQAs: Assay potency (e.g., API content), dissolution rate.	CQAs: Cell-based activity assays, target binding affinity, Fc-mediated functions.	Biologic activity depends on functional interactions (e.g., receptor binding), necessitating cell-based or functional assays.
Stability	CQAs: Degradation products (e.g., oxidation, hydrolysis), solubility.	CQAs: Protein aggregation, deamidation, fragmentation, charge variants.	Biologics are prone to post-translational modifications and physical instability due to their macromolecular nature.
Manufacturing Control	CQAs: Particle size, blend uniformity.	CQAs: Glycan profiles, charge heterogeneity, viral safety (e.g., clearance validation).	Biologic production involves living systems, introducing variability in post-translational modifications (e.g., glycosylation).
Case Example	Paracetamol: CQAs include crystal form (polymorphism) and dissolution profile.	Adalimumab: CQAs include charge variants (acidic/basic species) and glycosylation at Fc region.	Small-molecule CQAs focus on physicochemical consistency; biologics require monitoring of microheterogeneity.

**Table 3:** Fishbone Diagram of methodologies and tools for Quality by Design (QbD).

Category	Methodologies and Tools	Application and Purpose	Examples/References
1. Target Definition	QTPP (Quality Target Product Profile)	Define product quality goals (e.g., dosage, stability, pharmacokinetics).	ICH Q8(R2) guidelines; Case: Solid oral dosage QTPP for bioavailability control.
2. Risk Assessment	FMEA (Failure Mode Effects Analysis) FTA (Fault Tree Analysis)	Identify and prioritize risks to CQAs (e.g., raw material variability, process steps).	Tool: Ishikawa diagrams; Case: Biologic aggregation risk mitigation.
3. Experimental Design	DoE (Design of Experiments) RSM (Response Surface Methodology)	Optimize CPPs and CMAs through statistical modeling (e.g., formulation robustness).	Software: JMP^®^ 18, Minitab^®^ 20.3(60-bit); Case: Tablet hardness optimization.
4. Process Analytics	PAT (Process Analytical Technology) NIR Spectroscopy HPLC	Real-time monitoring and control of CPPs (e.g., blend uniformity, reaction completion).	FDA PAT Framework; Case: Continuous manufacturing of monoclonal antibodies.
5. Modeling and Control	Design Space Multivariate Analysis (MVA) Control Charts	Establish validated operating ranges and adaptive control strategies.	ICH Q10; Case: Design space for lyophilization cycle optimization.
6. Continuous Improvement	PDCA Cycle Six Sigma SPC (Statistical Process Control)	Reduce variability and enhance process robustness through iterative learning.	Case: Reducing batch failures in API synthesis via SPC.

Diagram Logic: Central Spine: QbD lifecycle (from QTPP to continuous improvement). Branches: key categories (Target Definition, Risk Assessment, etc.) with associated tools. Regulatory alignment: tools align with ICH Q8 and Q11, emphasizing science- and risk-based approaches. Critical tools: QTPP and CQAs drive target setting; DoE and PAT enable process understanding; Design Space ensures regulatory flexibility.

**Table 4:** QbD development workflow for solid oral dosage forms (e.g., tablets) ^1^.

Stage	Description	Key Outputs	Application Examples
1. Define QTPP	Establish target product quality attributes based on clinical and regulatory requirements.	QTPP document specifying attributes (e.g., dose strength, dissolution profile, stability).	Example ^2^: Immediate-release tablet targeting >85% dissolution within 30 min (pH 1.2–6.8).
2. Identify CQAs	Link critical quality attributes to safety, efficacy, and patient-centric performance.	Prioritized CQAs list (e.g., hardness, friability, disintegration time, content uniformity).	Case: Disintegration time as a CQA for fast-dissolving aspirin tablets.
3. Risk Assessment	Evaluate material attributes (CMAs) and process parameters (CPPs) impacting CQAs using FMEA/FTA.	Risk priority matrix, identified CPPs (e.g., granulation moisture, compression force).	Tool: Ishikawa diagram for root cause analysis of tablet capping.
4. Doe and Process Optimization	Conduct multivariate studies to optimize CMAs and CPPs (e.g., excipient ratios, granulation parameters).	Predictive models (e.g., RSM), validated process ranges.	Case: Optimization of binder concentration and compression force for acetaminophen tablets.
5. Establish Design Space	Define multidimensional ranges for CPPs and CMAs ensuring product quality.	Design space model with proven acceptable ranges (e.g., lubrication time: 3–5 min).	Regulatory Example: ICH Q8-compliant design space for sustained-release matrix tablets.
6. Control Strategy	Implement real-time monitoring (PAT) and procedural controls to mitigate variability.	Control plan (e.g., in-line NIR for blend uniformity, tablet hardness testing).	Case: Real-time release testing (RTRT) for metformin HCl tablets using Raman spectroscopy.
7. Continuous Verification	Monitor process performance and update strategies using lifecycle data.	Updated control limits, reduced batch failures, enhanced process capability (C_pk_ > 1.33).	Tool: Statistical Process Control (SPC) charts for tracking tablet weight variability.

^1.^ Critical parameters for tablets: CMAs: API particle size, excipient functionality (e.g., flowability of microcrystalline cellulose). CPPs: granulation endpoint (torque/power consumption), compression force, coating pan temperature. Analytical tools: dissolution testing (USP apparatus), friability testers, near-infrared (NIR) spectroscopy for content uniformity. Regulatory alignment: complies with ICH Q8 (Pharmaceutical Development), ^2^ Kim H. A. (2022) [82].

**Table 5:** Comparison between traditional quality testing and Quality by Design (QbD) methodology.

Aspect	Traditional Quality Testing	Quality by Design (QbD)
Quality Philosophy	Quality is tested into the product (reactive).	Quality is built into the product (proactive).
Focus	End-product testing to meet specifications.	Risk- and science-based process understanding to ensure quality during development.
Key Methods	Sampling and off-line testing (e.g., HPLC, dissolution testing).	Systematic tools: DoE, PAT, risk assessment, design space, and multivariate analysis.
Process Design	Fixed processes; limited flexibility.	Flexible design space with predefined operating ranges.
Control Strategy	Relies on batch-wise inspection and acceptance criteria.	Real-time monitoring (PAT) and adaptive controls to mitigate variability.
Data Utilization	Retrospective analysis of quality data.	Predictive modeling and continuous process verification.
Risk Management	Reactive identification of failures (post-production).	Proactive risk assessment (e.g., FMEA) to prioritize and mitigate risks early.
Regulatory Flexibility	Rigid; changes require regulatory re-approval.	Supports post-approval changes within the design space (ICH Q8–Q11 compliance).
Cost Efficiency	Higher long-term costs due to rework, scrap, and recalls.	Reduced lifecycle costs via optimized processes and fewer deviations.
Product Understanding	Limited understanding of process-product relationships.	Deep scientific understanding of critical material attributes (CMAs) and CPPs.
Innovation	Minimal emphasis on process improvement.	Encourages continuous improvement and innovation through iterative learning.

**Table 6:** Challenges in global regulatory harmonization: divergent perspectives on design space (case study of FDA vs. EMA).

Aspect	FDA (U.S.)	EMA (EU)	Harmonization Gaps and Implications
Regulatory Framework	Emphasizes *risk-based flexibility* under ICH Q8-Q11. Supports *post-approval changes* within design space without prior approval.	Stricter *predefined data requirements* for design space validation. Requires prior approval for major changes, even within design space.	Conflict: FDA’s “enabled flexibility” vs. EMA’s *cautionary approach* delays global dossier alignment.
Design Space Acceptance	Encourages *broader design spaces* with multivariate interactions (e.g., dissolution and compression). Case: Approved design space for a modified-release tablet with 3 CPPs.	Prefers *narrower, parameter-specific ranges (e.g., granulation* moisture as standalone CPP). Case: Rejected a design space model due to insufficient interaction data.	Impact: Sponsors must generate region-specific data, increasing R&D costs.
Change Management	Allows *real-time monitoring* (PAT) to justify adjustments within design space (e.g., blend uniformity via NIR).	Demands *extensive stability data* for post-design space changes (e.g., 12-month real-time stability).	Delay: EMA’s data requirements prolong time-to-market for multinational products.
Data Requirements	Accepts *mechanistic models* (e.g., PBPK) to support design space boundaries.	Prioritizes *empirical data* over predictive models for biologics (e.g., mAb aggregation risks).	Inconsistency: Model-informed approaches face EMA skepticism, hindering innovation adoption.
Communication	Open dialog via *QbD pilot programs* and pre-submission meetings.	Relies on *formal scientific advice* with limited iterative feedback.	Barrier: Asymmetric communication channels complicate global strategy alignment.

Case examples: Sections in Regulatory Information and Dissolution Methods Database, Food and Drug Administration (.gov): approved a design space for oncology tablets integrating dissolution and hardness CPPs (2019). EMA: rejected a biosimilar design space due to insufficient glycosylation control data (2021).

**Table 7:** Typical QbD tools and their applications in pharmaceutical development and manufacturing.

QbD Tool	Description	Application Scenarios
Risk Assessment (RA)	Systematic identification and prioritization of risks affecting product quality.	Identifying critical process parameters (CPPs) and material attributes during development.
Design of Experiments (DoE)	Statistical approach to optimize processes by studying interactions between variables.	Screening and optimizing formulation variables, process conditions, and robustness testing.
Process Analytical Technology (PAT)	Real-time monitoring and control of manufacturing processes using analytical tools.	Ensuring consistent product quality through in-line or on-line measurements (e.g., spectroscopy).
Quality Target Product Profile (QTPP)	A prospective summary of quality characteristics for a drug product.	Defining target product attributes (e.g., dissolution rate, stability) in early development.
Critical Quality Attributes (CQAs)	Quantifiable properties or characteristics linked to product safety, efficacy, and performance.	Identifying parameters requiring tight control (e.g., impurity levels, tablet hardness).
Control Strategy	A plan to ensure process performance and product quality through monitoring and adjustments.	Mitigating variability in commercial manufacturing (e.g., sampling plans, feedback loops).
Design Space	Multidimensional combination of input variables proven to ensure product quality.	Defining validated operating ranges for scale-up and post-approval changes.
Failure Mode and Effects Analysis (FMEA)	Proactive risk assessment to prioritize failure modes based on severity, occurrence, and detectability.	Evaluating equipment reliability, process steps, or formulation stability risks.
Continuous Improvement (CI)	Iterative methodologies (e.g., PDCA cycle) to enhance process robustness and quality outcomes.	Reducing deviations, waste, and costs in long-term manufacturing.
Multivariate Data Analysis (MVDA)	Advanced statistical techniques to interpret complex datasets from multiple variables.	Root cause analysis of process deviations or batch failures.

**Table 8:** Categorization of QbD Tools Across Pharmaceutical Development Stages.

QbD Stage	Key Activities	Associated Tools/Methodologies
1. Product Design	- Conceptualization and research for consistent quality	- Critical Quality Attributes (CQAs) definition - Design of Experiments (DOE) - Multivariate analysis - Process Analytical Technology (PAT) (real-time monitoring of dissolution, etc.)
2. Process Design and Optimization	- Scalable manufacturing process development - Process synthesis (continuous, batch, semi-batch) - Process modeling (CPP definition) - Real-time control and risk mitigation	- PAT integration (CQA/CPP monitoring) - Failure Mode and Effects Analysis (FMEA) - Hazard Analysis and Critical Control Points (HACCP) - Model Predictive Control (MPC) (real-time optimization)
3. Continuous Improvement and Control	- Ongoing optimization via real-time data feedback - Adaptive control for process variations	- PAT-driven data analytics - Continuous monitoring systems - Adaptive control strategies

## Abbreviations

QbD	Quality by Design
CQAs	Critical Quality Attributes
TPQP	Target Product Quality Profile
DoE	Design of Experiments
PAT	Process Analytical Technology
QC	Quality Control
HPLC	High-Performance Liquid Chromatography
CPPs	Process Parameters
ICH	International Council for Harmonisation
CMAs	Critical Material Attributes
FMEA	Failure Mode and Effects Analysis
QMS	Quality Management Systems
PROs	Patient-Reported Outcomes
RWE	Real-World Evidence
PFDD	Patient-Focused Drug Development
IVIVC	In Vitro-in Vivo Correlations
NIR	Near-Infrared Spectroscopy
ADCC	Alter antibody-Dependent Cellular Cytotoxicity
HCP	Host Cell Protein
SEC	Size Exclusion Chromatography
CE	Capillary Electrophoresis
PLS	Partial Least Squares
RSM	Response Surface Methodology
MVA	Multivariate Analysis
PCA	Principal Component Analysis
RTRT	Real-Time Release Testing
MPC	Model Predictive Control
CCPs	Critical Control Points
ANNs	Artificial Neural Networks
DNNs	Deep Neural Networks
RL	Reinforcement learning
PSD	Particle Size Distribution
CM	Continuous Manufacturing
OOS	Out-of-Specification
CHO	Chinese Hamster Ovary
MOI	Multiplicity of Infection
HA	Hemagglutinin
ATMPs	Advanced Therapy Medicinal Products
SLS	Selective Laser Sintering
FDM	Fused Deposition Modeling
BCS	Biopharmaceutics Classification System
